# Ethnicity and wealth: The dynamics of dual segregation

**DOI:** 10.1371/journal.pone.0204307

**Published:** 2018-10-10

**Authors:** Anand Sahasranaman, Henrik Jeldtoft Jensen

**Affiliations:** Department of Mathematics and Centre for Complexity Science, Imperial College London, London, United Kingdom; Universidad Veracruzana, MEXICO

## Abstract

Creating inclusive cities requires meaningful responses to inequality and segregation. We build an agent-based model of interactions between wealth and ethnicity of agents to investigate ‘dual’ segregations—due to ethnicity and due to wealth. As agents are initially allowed to move into neighbourhoods they cannot afford, we find a regime where there is marginal increase in both wealth segregation and ethnic segregation. However, as more agents are progressively allowed entry into unaffordable neighbourhoods, we find that both wealth and ethnic segregations undergo sharp, non-linear transformations, but in opposite directions—wealth segregation shows a dramatic decline, while ethnic segregation an equally sharp upsurge. We argue that the decrease in wealth segregation does not merely accompany, but actually drives the increase in ethnic segregation. Essentially, as agents are progressively allowed into neighbourhoods in contravention of affordability, they create wealth configurations that enable a sharp decline in wealth segregation, which at the same time allow co-ethnics to spatially congregate despite differences in wealth, resulting in the abrupt worsening of ethnic segregation.

## Introduction

The UN predicts that global urban population will grow by an estimated 2.5 billion from now until 2050 [1]. We are already seeing the increasing intensification of inequality in the form of wealth-based and ethnicity-based segregations in cities across the world. The UN’s Sustainable Development Goals explicitly call for cities to be made inclusive and safe [2]. Making cities inclusive requires meaningful responses to the underlying challenges of inequality and segregation.

The Cambridge dictionary defines segregation [3] as the phenomenon of keeping one group of people apart from another and treating them differently, especially because of race or sex. In this paper, we focus on two kinds of urban segregation: wealth segregation, which is the spatial separation of rich and poor, and ethnic segregation, which is the spatial separation of people by ethnicity (including race, caste, religion, ethnic origin or language).

We had previously investigated the dynamics underlying the emergence and reversal of wealth based segregation in cities [4] as well as the longer term evolution of economic status of urban neighbourhoods over time [5]. The basis of this work is the Schelling model [6], the classic framework to study segregation. It explains the emergence of racial segregation on the basis of interactions of individual agent preferences for ‘like’ neighbours, with even small preferences for like neighbours at an individual level leading to emergence of segregated patterns at a collective level. The striking finding of the Schelling model is that even if all agents have a high tolerance for agents unlike themselves (up to two-thirds of their neighbours), large scale segregation ensues. The Schelling model is robust across parameter specifications such as neighbourhood shape and size, heterogeneity of agent preferences [7] [8] [9] [10], as well as agent choice functions, though this is a matter of some debate [11] [12]. Bruch and Mare [11] argue that agents responding to even small changes in neighbourhood composition yields substantially diminished segregation, but van de Rijt, Siegel, and Macy [12] contradict Bruch and Mare, finding that even in the case of continuous functions, the tendency to segregate is pronounced. They point out that only in the case of neighbourhood choice being sufficiently random, with agents often moving into non-preferred destinations, does diminished segregation obtain. Physicists have implemented physical analogues of the Schelling model [13] [14], linking it to the phenomenon of clustering of physical particles. Stauffer and Solomon [15] have studied the Schelling process as an Ising model [16], observing a phase transition between segregated and mixed configurations. Recent research [17] [18] has also explored the interface between segregation and de-segregation, with potential mechanisms to precipitate de-segregation.

We had modeled wealth based segregation [4], modifying the Schelling model [6] by replacing agent tolerance levels with neighbourhood wealth threshold levels (based on the wealth profile of extant agents in the neighbourhood) that agents were required to satisfy in order to be able to move into these neighbourhoods. We found a sharp transition from segregated to mixed wealth equilibrium when only a small fraction of agents were allowed to move into neighbourhoods in contravention of the neighbourhood wealth condition. We argue that the sharp transformation is caused by an entropic effect whereby there is a non-linear increase in the number of moves where threshold condition is satisfied (also called allowed moves) as a function of even a small number of moves where threshold condition is contravened (also called Disallowed-Realised moves). This effect appears to be consistent with the empirical findings of Benenson, Hatna, and Or [19], whose work on Israeli cities suggests that the introduction of a small number of highly tolerant agents could result in significantly mixed residential patterns. It is important to note that there is a substantial difference in interpretation of the observed phenomenon, with our work positing the mixed equilibrium as an outcome of even a limited contravention of wealth threshold conditions, while Benenson, Hatna, and Or present a classical Schelling interpretation of the phenomenon based on agent tolerances.

In the classic Schelling model with agents defined by their tolerance level towards non co-ethnics, the emergence of ethnic segregation is a robust result. When agent movement is dictated by their wealth, and in the absence of any external intervention, wealth-based segregation ensues. The interaction between these two segregations—wealth segregation and ethnic segregation—remains an area that has received little attention in the literature. Given this context, we seek to investigate the dynamics of agent movement in a city based on the interactions between wealths and ethnicities of agents. We are interested in studying the potential emergence of ‘dual’ segregation—namely, ethnic segregation and wealth-based segregation.

There has been some prior work on the interaction between economic ability and fixed cultural traits of agents in determining residential choice, which has revealed that these two determinants of agent mobility can be reinforcing in certain scenarios and offsetting in others [20] [21]. Bruch [20] studied the effect of income inequality within and between races on racial segregation, and found that when within-group income inequality is low, increase in between-group inequality yields increase in racial segregation, but that when within-group inequality is high, increase in between-group inequality results in much smaller effects on racial segregation. Bruch concludes that this small effect of income inequality is due to offsetting effects at the high and low ends of the income distribution. Depending on extent of income inequality between and within races, and the relative size of minority race population, the offsetting mechanism could result in decreased, increased, or constant racial segregation. Therefore, while Bruch’s work focuses on the impact of income distributions (between and within races) on racial segregation, it does not examine the phenomenon of income segregation itself, and it is indeed this interaction between dual segregations that drives our work. Also, agent behaviour in Bruch’s simulated model is estimated from Panel Study of Income Dynamics (PSID) data, while our model uses the Schelling model as the base, with simple heuristics guiding agent actions (described in next section). The notion of dual segregations has however received attention in the work of Benard and Willer [21], who examine the concomitant emergence of both wealth- and status- based segregation. The status of a neighbourhood is determined by the status of agents that live there—status being a measure of the desirability of that neighbourhood. Agents attempt to move into the highest status neighbourhood their wealth can afford at each step. When there is no correlation between status and wealth, and housing price endogeneity increases, Benard and Willer find a trade-off—wealth segregation increases but status segregation marginally decreases. Our model focuses only on complete housing price endogeneity, with no exogenous pricing effects. When neighbourhood housing prices are endogenously determined, Benard and Willer find that wealth segregation and status segregation are found to progressively increase as status-wealth correlation increases. While our focus is on ethnicity (a discrete variable) as opposed to status (a continuous variable), we would still expect that this result will be replicated in our study as well. However, our objective is not only to study the emergence of dual segregations based on the correlation between wealth and ethnicity, but most importantly, to examine mechanisms that enable their reversal. Specifically, we seek to understand the nature of the relationship between these dual segregations and its implications as we progressively allow agents to move into neighbourhoods they cannot afford.

An additional aspect that has largely been neglected is the question of migration into a Schelling framework. While the process of migration itself has been modeled on the basis of social network structure binding individuals [22], Urselmans [23] appears to be the first instance of exploring the effect of migration on racial segregation. In Urselmans’ model, the migrating population belongs to a different racial group from the extant population of the city, and it emerges that overall agent happiness in terms of population of neighbourhood co-ethnics converges, irrespective of the size and rate of migration.

It is the spirit of the work of Benard and Willer [21] as well as Urselmans [23] and Bruch [20] that we attempt to extend in our simple model to study the effect of migrating agents of a different ethnicity into a city, on the emergence of and interaction between wealth and ethnic segregation. The specific contributions we hope our model will make are: (i) in exploring the dynamics that result due to the progressive contravention of the affordability condition (allowing agents to move into neighbourhoods they cannot afford) on the relationship between the two types of segregation; (ii) in exploring the impact of continuous migration of agents of a different ethnicity into a ‘city’ of co-ethnic residents on the two types of segregation; (iii) in making agents’ choice of movement contingent upon both the wealth and ethnic composition of neighbourhoods; and (iv) in enabling stochasticity in agent movement as opposed to purely deterministic rules to capture some of the randomness inherent in human agent actions and their impact on the dual segregations.

## Model definition and specifications

We consider a ‘city’ with *M* neighbourhoods, and each neighbourhood *i* (*i* ∈ 1, …, *M*), is populated by *P*(*i*) agents (households). Initially, agents are distributed equally across each of the M neighbourhoods, such that *P*(*i*) = *P*(*j*) for 1 ≤ *i*, *j* ≤ *M*. This model follows in the tradition of earlier metapopulation models [24] [25], where each location is a neighbourhood with a carrying capacity, as opposed to the classic Schelling model where each location in a lattice represents a single agent. However, our model also differs significantly from these models in that the dynamics are driven both by ethnicity and wealth considerations.

All the agents that populate the city initially are co-ethnics, ie. they belong to the same ethnicity, *e*_0_. Every agent has two attributes—wealth and tolerance level. We sample agent wealths from a normal distribution—*N*(10, 1). The total wealth of each neighbourhood *i* is the sum of individual agent wealths and is denoted by *W*_tot_(*i*). We define the tolerance level (*τ*) as the fraction of non co-ethnic population in a neighbourhood that an ethnic *e*_0_ agent is happy to tolerate. Sethi and Somanathan [26] state that studies on preferences for neighbours have historically shown that people prefer some degree of integration with a bias for own-type neighbours. They point, for instance, to a survey of 8,500 households on urban inequality from the 1990s [27] which finds that, on average, African Americans preferred neighbourhoods with 40% like neighbours, while white Americans preferred neighbourhoods with 52% white neighbours. We set *τ* = 0.5 for all the initial co-ethnic *e*_0_ agents in our simulations.

Given this initial set up, the dynamics of the model now incorporates the entry of the other ethnicity, *e*_1_, into the city. At each iteration of the model, a certain number of agents of ethnicity *e*_1_, defined as a fraction of extant city population, attempt to enter the city. For our simulations, we set this fraction, *r*_mig_ = 0.005. Given *r*_mig_ and the total population of the city at the end of the previous iteration (*P*_t-1_), the number of agents attempting to migrate into the city at time step *t*, *Mig*_t_, is determined as (Eq 1):

$$\begin{array}{c} {\text{Mig}}_{t}=r_{mig}P_{t-1} \end{array}$$ (1)

Based on this, at any given time t, we have a total of *Mig*_t_ agents attempting to enter the city, but the actual number that are able to enter the city is contingent upon the wealth of the agent and the population of *e*_1_ co-ethnics in city neighbourhoods. While the salience of wealth of the agent on its ability to afford entry into the city is fairly obvious, the dependency of entry on extant co-ethnic populations is supported by both theoretical and empirical work on mechanisms of migrant entry into cities. The theory of spatial assimilation [28], for instance, posits that immigrants first settle in segregated ethnic enclaves both due to the comfort of being amongst co-ethnics and because of the inability of many newcomers to afford living in neighbourhoods of the majority ethnicity. The theory further contends that as their socioeconomic condition improves, migrants seek to improve their spatial location and move into wealthier neighbourhoods of the dominant ethnic majority. Large international cities like London have distinct neighbourhoods for new immigrants and detailed ethnographies confirm that despite overall being an ethnically diverse city, different ethnicities are concentrated by area, with migration being a primary contributor [29].

It is likely that the wealth of incoming co-ethnics (*e*_1_) is, on average, lower than that of the dominant ethnic (*e*_0_) inhabitants. We base this on evidence from across a number of contexts. For instance, in the United States, data shows that median wealth and incomes of white American households are 5.3 times [30] and 1.6 times [31] those of African American households. In India, it has been found wealth is stratified by caste, with historically disadvantaged castes—the Scheduled Castes and Scheduled Tribes—being economically worse off than all other caste groups, encapsulated by the fact that in urban India the median income of other caste households is 2.1 times that of disadvantaged caste households [32]. This phenomenon is also evidenced in the forced migration of predominantly Muslim Syrian refugees into Europe as a result of the Syrian conflict. A socioeconomic survey of Syrian refugees finds that most refugees worked in low-paying and insecure jobs, evidence of their precarious economic condition and low wealth status [33]. Given this evidence, we sample agent wealths for incoming *e*_1_ agents from a normal distribution with lower mean wealth than the wealth distribution for *e*_0_ agents—*N*(7, 1).

For each amongst the *Mig*_t_ *e*_1_ agents (*W*^A^) whose wealth is greater than or equal to the median wealth of a randomly chosen city neighbourhood *i* ($W_{\text{Med}}^{\text{i}}$), entry into that neighbourhood is guaranteed—ie. probability of agent entry, *p*_entry_ = 1. In case of the remaining agents for whom the wealth threshold condition is not satisfied, entry into the city is stochastically determined, based on the ratio of population of *e*_0_ agents ($P_{\text{i}}^{{\text{e}}_{0}}$) to total population ($P_{\text{i}}^{\text{Tot}}$) in the randomly chosen cell *i* (as specified in Eq 2). The calculated value of *p*_entry_ is compared to a random number generated between 0 and 1, and if the random number is lesser than *p*_entry_, then the agent enters the city.

$$\begin{array}{c} p_{\text{entry}}=\{\begin{array}{cc} 1, & \text{if}\;W^{A}\geq W_{Med}^{i}\\ \exp(-\beta_{in}\frac{P_{i}^{e_{0}}}{P_{i}^{Tot}}), & \text{otherwise} \end{array} \end{array}$$ (2)

**Fig 1:**
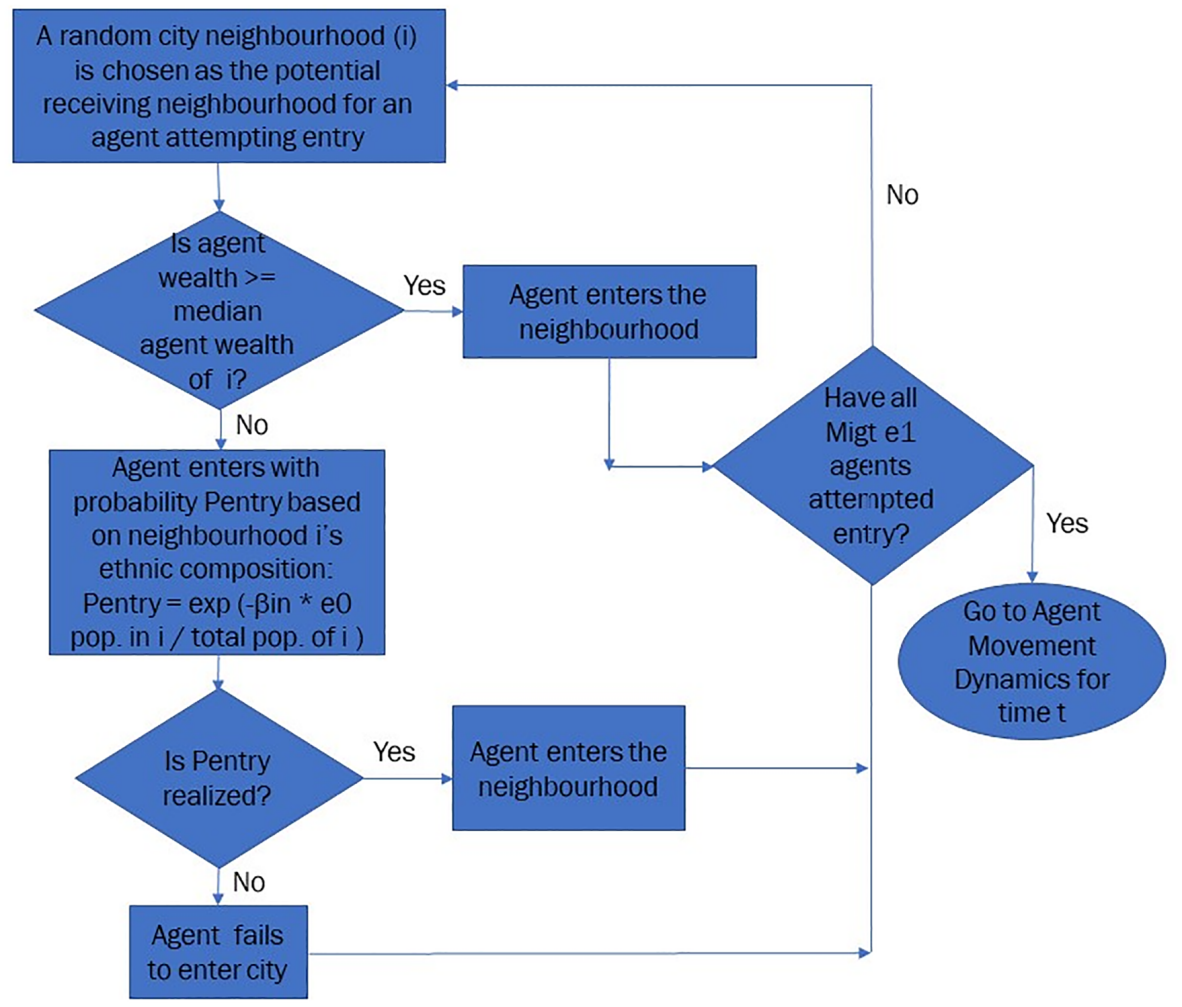
Flowchart: Mechanics of agent entry.

The probability of an *e*_1_ agent being able to enter a neighbourhood increases with decreasing proportion of *e*_0_ agents in a neighbourhood, even when in contravention of the wealth threshold condition. In Eq 2, *β*_in_ is simply a calibrating factor that determines *p*_entry_. In the limit *β*_in_ → ∞, the probability of *e*_1_ agent entry becomes purely dependent on satisfaction of the wealth threshold condition, while in the limit *β*_in_ → 0, all potential *Mig*_t_ *e*_1_ agents enter the city. For the simulations of the model, we choose *β*_in_ = 1. Fig 1 depicts a flowchart describing the mechanics of agent entry:

Once all eligible migrants have entered the city for a given iteration (time step *t*), the population of the city (*P*_t_) is the sum of the population of agents at time step *t* − 1, *P*_t-1_, and the new migrant population that has successfully entered at time step *t*. After the dynamics of e_1_ agent entry are completed for an iteration (time step *t*), we shift focus to agent movement within the city for that iteration.

Agent movement within the city occurs as a two step process—first, the decision of an agent in choosing to move from or stay in its current location and second, if it does choose to move, the ability of the agent to accomplish the move given its wealth. The agent decision itself involves two components—comparison of neighbourhood wealth profiles and comparison of neighbourhood ethnicity profiles.

At time step *t*, we randomly choose *P*_t_ agents sequentially, and each of them then makes the choice of moving within the city. The choice of *P*_t_ agents at *t* ensures that, on average, every agent has the opportunity to decide on moving at each time step. Each agent makes the first part of its decision to stay or move based on the assessment of the wealth of its current and potential locations. Defining the decision to move based on neighbourhood wealth comparison is supported by empirical evidence on the link between the economic status of neighbourhoods and socioeconomic outcomes, as discussed in the previous section. Our previous work [5] also finds that the use by agents of a neighbourhood comparison heuristic to determine their movement decision is critical to generating long term patterns of empirically observed change in the economic status of neighbourhoods in cities. For each such randomly chosen agent, *A*, in neighbourhood *i*, a random receiving cell *j* is chosen and the neighbourhood comparison condition is applied—that is, the median wealths of these neighbourhoods *i* and *j* are compared. If the median wealth of the receiving cell *j* ($W_{\text{Med}}^{\text{j}}$) is greater than or equal to that of the agent’s current location *i* ($W_{\text{Med}}^{\text{i}}$), then the agent chooses to attempt a move from its current location, ie. *p*_init_choice_ = 1. If the median wealth of *i* is greater than that of *j*, the agent would, in general, prefer to stay in its current location, but we model an element of stochasticity in this choice—to capture other aspects that might influence such a decision (Eq 3). For instance, this stochasticity could capture the conflict inherent in weighing the relative merits of neighbourhood wealth profiles and neighbourhood ethnicity profiles in choosing to stay or attempting to move.

$$\begin{array}{c} p_{\text{init}\_\text{choice}}=\{\begin{array}{cc} 1, & \text{if}\;W_{Med}^{j}\geq W_{Med}^{i}\\ \exp(-\beta_{choice}\left(W_{Med}^{i}-W_{Med}^{j}\right)), & \text{otherwise} \end{array} \end{array}$$ (3)

*β*_choice_ is the calibrating factor that determines *p*_init_choice_. In the limit *β*_choice_ → ∞, choice is completely deterministic as the only agents that choose to attempt a move are those for whom $W_{\text{Med}}^{\text{j}}\geq W_{\text{Med}}^{\text{i}}$. In the limit *β*_choice_ → 0, choice is again completely deterministic as all agents choose to attempt a move. For our model, we choose *β*_choice_ = 10, to allow for some minimal movement in breach of the neighbourhood comparison condition.

The second step in the agent’s decision making process requires an assessment of the ethnicity of agents in its current and receiving cells. We now incorporate the ethnicity based effects, as in the classic Schelling model, for the *e*_0_ agents who are defined to have a migrant tolerance level of *τ*. We do not model a contrasting tolerance level for the incoming *e*_1_ agents in view of our earlier discussion on the lower wealth status, on average, of immigrants and their desire to move into the better neighbourhoods of the dominant community over time.

Given that *p*_init_choice_ is realised for a randomly chosen *e*_0_ agent, the next comparison is between the fraction of *e*_1_ agents in the two cells. If the fraction of *e*_1_ agents in the receiving cell ($f_{1}^{\text{j}}$) is lesser than either *τ* or the fraction of *e*_1_ agents in the agent’s current cell ($f_{1}^{\text{i}}$), then the agent chooses to attempt a move with probability 1, ie. *p*_final_choice_ = 1. If, however, the fraction of *e*_1_ agents in the receiving cell is greater than both the fraction of *e*_1_ agents in its current location and *τ*, *p*_final_choice_ = 0 (Eq 4). For all *e*_1_ agents seeking to move based on the realisation of *p*_init_choice_, *p*_final_choice_ = 1 (Eq 5).

$$\begin{array}{c} p_{\text{final}\_\text{choice}}\left(e_{0}\right)=\{\begin{array}{cc} 1, & \text{if}\;f_{1}^{i}\geq f_{1}^{j}\;\text{or}\;\tau \geq f_{1}^{j}\\ 0, & \text{otherwise} \end{array} \end{array}$$ (4)

$$\begin{array}{c} p_{\text{final}\_\text{choice}}\left(e_{1}\right)=1 \end{array}$$ (5)

Finally, if *p*_final_choice_ is realised and the agent has chosen to attempt a move out of its current location, then the actual occurrence of the move is determined by the agent’s wealth. If the agent’s wealth (*W*_A_) is greater than or equal to the median wealth of the receiving cell ($W_{\text{j}}^{\text{Med}}$), then the agent moves to the receiving neighbourhood with probability *p*_move_ = 1. If the wealth threshold condition is not satisfied, the agent move becomes stochastic, with probability *p*_move_ (Eq 6):

$$\begin{array}{c} p_{\text{move}}=\{\begin{array}{cc} 1, & W_{A}\geq W_{j}^{\text{Med}}\\ \exp(-\beta_{move}\left(W_{j}^{\text{Med}}-W_{A}\right)), & \text{otherwise} \end{array} \end{array}$$ (6)

**Fig 2:**
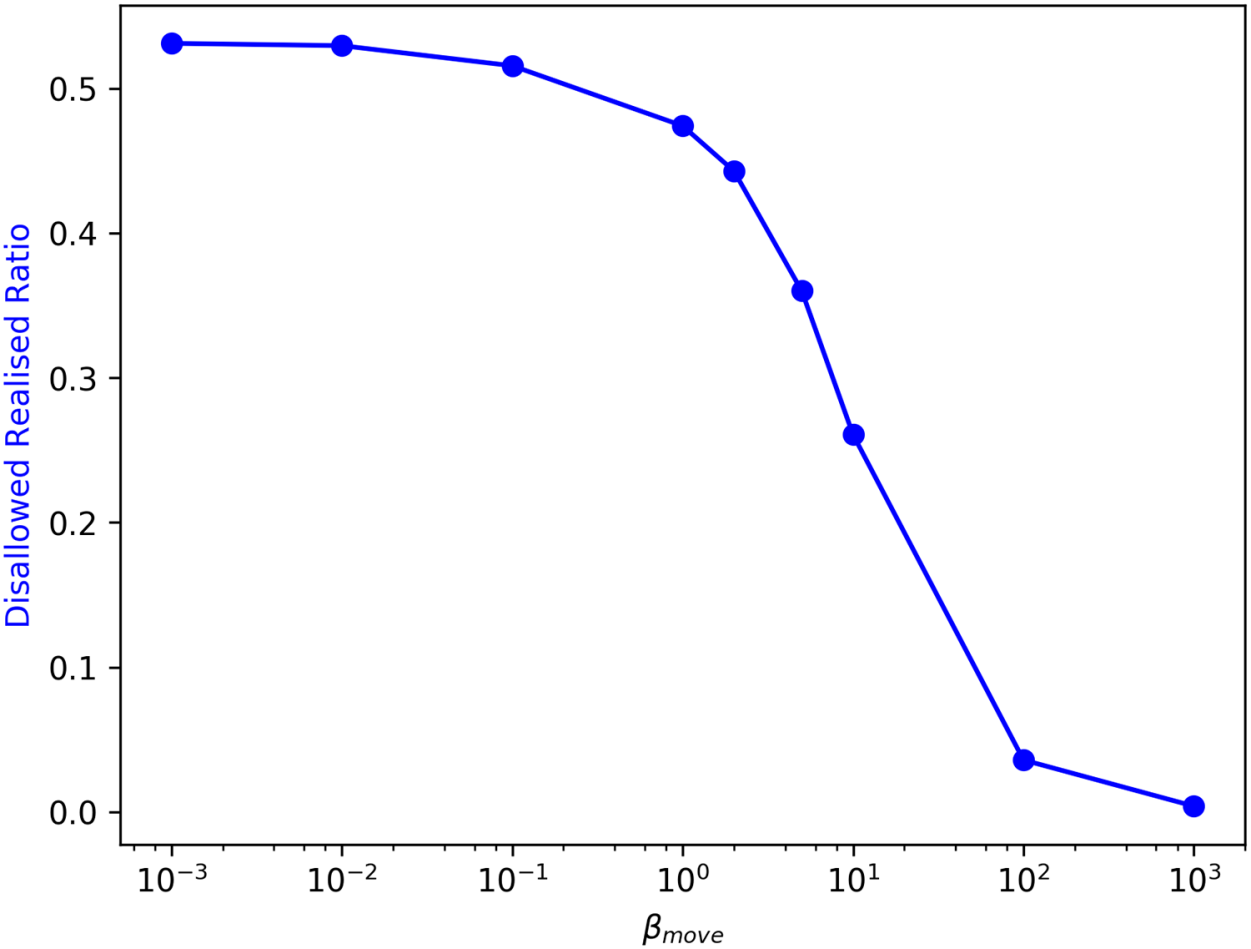
Change in Disallowed-Realised ratio with *β*_move_.

*β*_move_ is the calibrating factor that determines *p*_move_. In the limit *β*_move_ → ∞, movement is completely deterministic as the only moves that occur are those that satisfy the threshold wealth condition. As we explored earlier [4], decreasing *β*_move_, thereby enabling moves in contravention of the threshold wealth condition, yields a sharp transformation from a highly wealth segregated to a mixed wealth configuration even at a very small fraction of such moves. These moves in contravention of the wealth threshold are termed as Disallowed-Realised moves, and the fraction of total Disallowed-Realised moves to total number of move attempts by agents is defined as the Disallowed-Realised ratio. Fig 2 describes the relationship between Disallowed-Realised ratio and *β*_move_.

**Fig 3:**
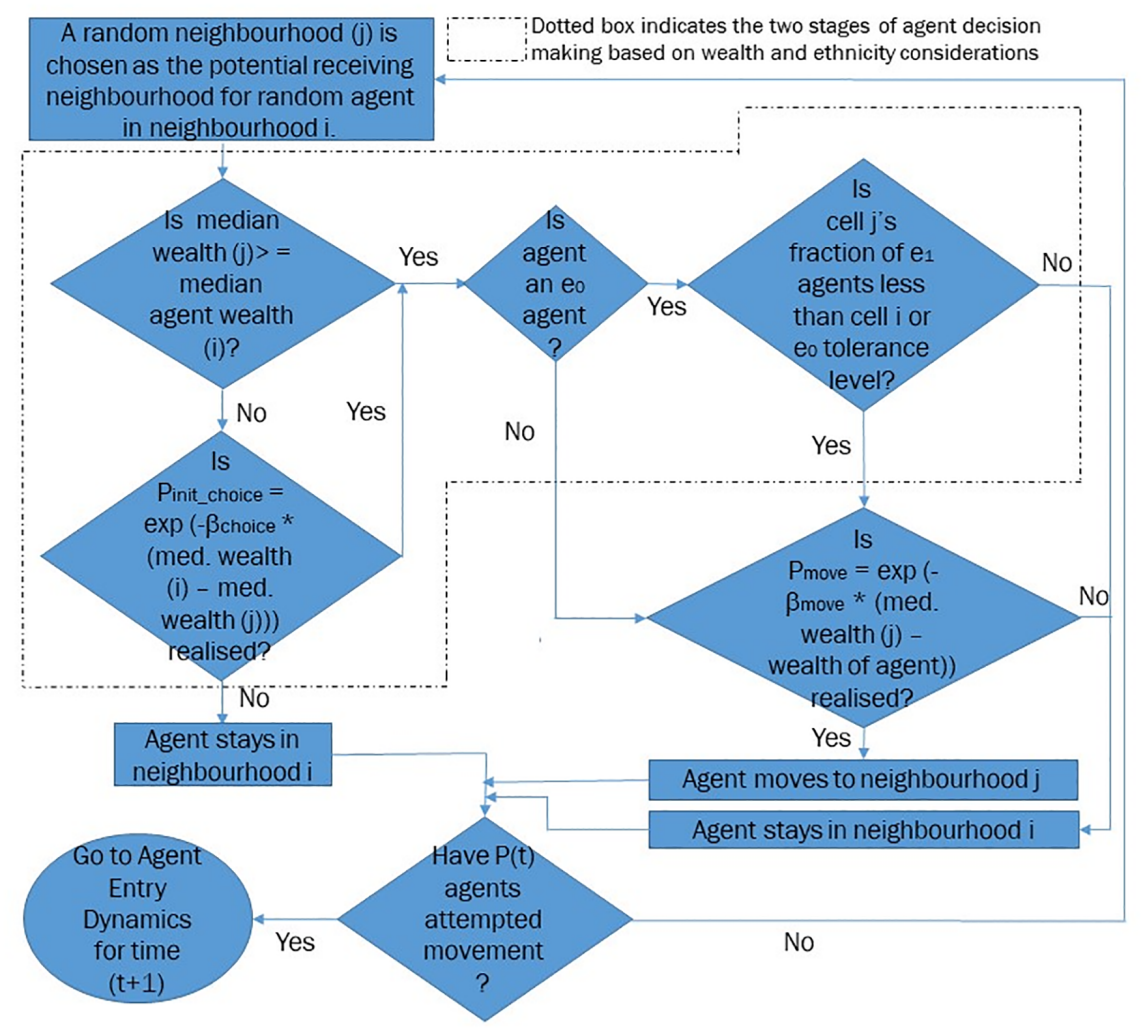
Flowchart: Mechanics of agent movement.

Disallowed-Realised moves could be thought of as public policy interventions (such as income support or vouchers) that enable households to move into neighbourhoods that they otherwise cannot afford. We now seek to study the impact on ethnic segregation of a progressively decreasing *β*_move_, while also verifying whether the sharp transformation from wealth segregated to mixed wealth states still occurs as previously observed. For this analysis, *β*_move_ takes the following values: 1000, 100, 10, 5, 1, 0.1, 0.01, and 0.001. Fig 3 depicts a flowchart describing the mechanics of agent movement within the city:

The update process in the model is sequential, which means that all model variables are updated at the end of each agent action. We initialise the city with *M* = 50 neighbourhoods and a total of 2,500 *e*_0_ agents. The model is run till it attains a dynamic equilibrium, where the aggregate level measures of ethnic- and wealth-segregation (described below) do not change, even though agent movement may still occur. The dynamics are run over 500 iterations, which means that each *e*_0_ agent, on average, is sampled 500 times, and all *e*_1_ agents, on average, are sampled as many times as the number of time steps (iterations) they spend in the city. We find that a dynamic equilibrium is well established by the 500^th^ iteration of the model, essentially meaning that the statistical properties of interest have stabilised by this time and do not undergo further change even though agent movement still occurs. 500 iterations of the model correspond to between 3 and 4.5 million simulations of individual agent choices. This range is due to the difference in the number of agents entering the city at each iteration, given the values chosen for the different calibrating parameters—*β*_in_, *β*_choice_, and *β*_move_. Finally, for each value of *β*_move_ detailed earlier, we run an ensemble of 25 runs of the entire model (Table 1).

**Table 1:** Model Parameters.

Parameter	Value
Number of Neighbourhoods (*M*)	50
Number of *e*_0_ Agents	2,500
Tolerance Level of *e*_0_ Agents (*τ*)	0.5
Entry Attempt Rate (*r*_mig_)	0.005 per iteration
Agent Wealth Distribution	*e*_0_ Agents: *N*(10, 1); *e*_1_ Agents: *N*(7, 1)
Number of Iterations	500
*β*_in_	1
*β*_choice_	10

Given our objective of understanding the potential emergence of ‘dual’ segregations, we measure the following outcomes at the end of the 50 time sweeps: Average Fraction of Rich Neighbours (*F*_R_) and Average Size of *e*_0_ Neighbourhoods (*S*_0_).

Average Fraction of Rich Neighbours (*F*_R_) is simply a measure of the average fraction of rich neighbours of a rich agent. A rich agent is defined as an agent with wealth in the top 15% of the population. For each rich agent *a*_R_, out of a total of *N*_R_ rich agents, we compute the number of its rich neighbours (*V*_R_(*a*_R_)) as a fraction of its total neighbuors (*V*(*a*_R_)) and average this quantity across all *N*_R_ rich agents (Eq 7).

$$\begin{array}{c} F_{R}=\frac{1}{N_{R}}\sum_{a_{R}=1}^{N_{R}}\frac{V_{R}\left(a_{R}\right)}{V(a_{R})} \end{array}$$ (7)

Average Size of *e*_0_ Neighbourhoods (*S*_0_) is a measure of the average number of *e*_0_ agents that are neighbours of *e*_0_ agents. For each agent of ethnicity *e*_0_, *a*_0_, out of the total *N*_0_ agents of ethnicity *e*_0_, we compute the number of its neighbours that are also *e*_0_ agents (*V*_0_(*a*_0_)) and average this across all *N*_0_ agents (Eq 8):

$$\begin{array}{c} S_{0}=\frac{1}{N_{0}}\sum_{a_{0}=1}^{N_{0}}V_{0}\left(a_{0}\right) \end{array}$$ (8)

## Results

**Fig 4:**
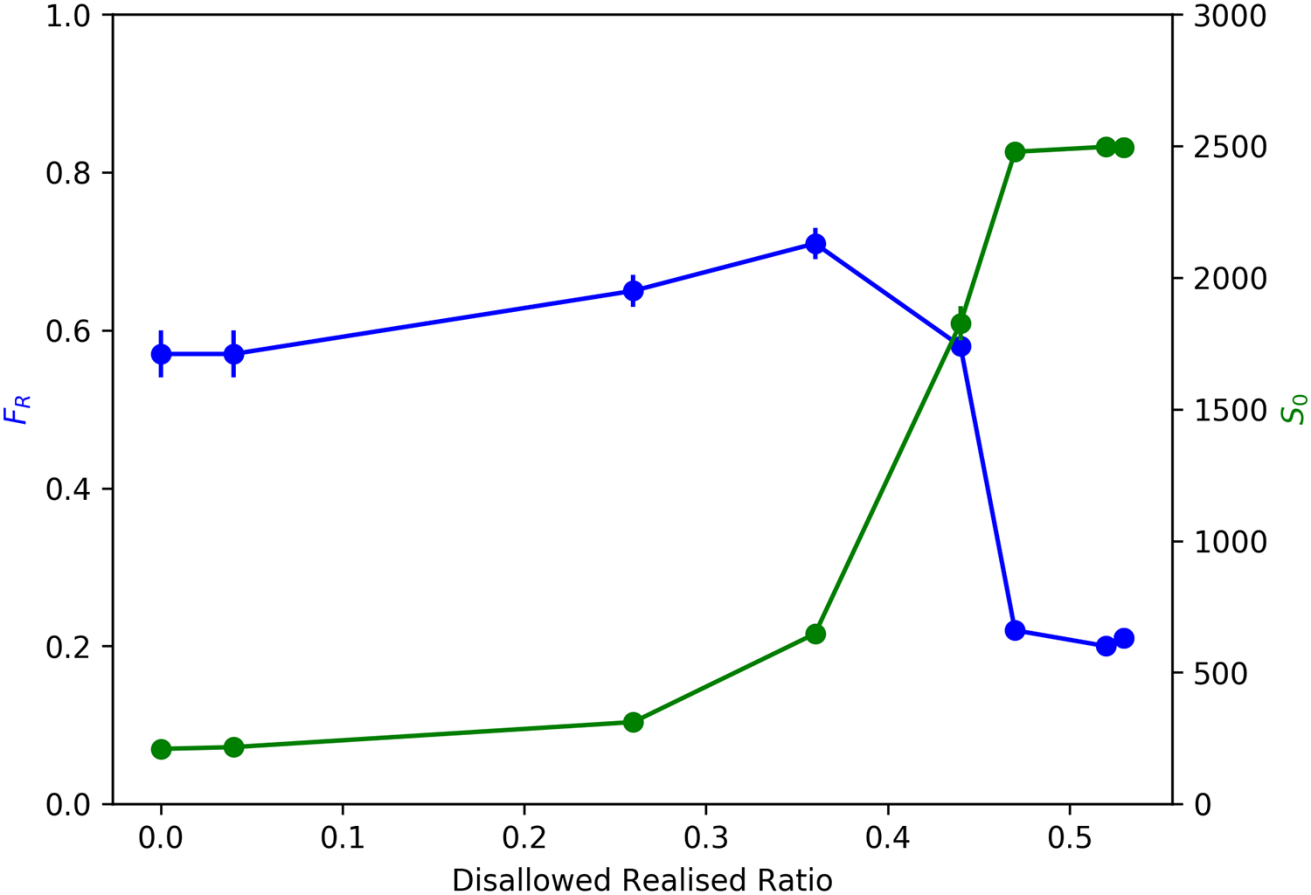
Emergence of ‘dual’ segregations. Blue: Average Fraction of Rich Neighbours (*F*_R_) v. Disallowed-Realised Ratio. Green: Average Size of *e*_0_ Neighbourhoods (*S*_0_) v. Disallowed-Realised Ratio. Error bars represent 95% Confidence Interval.

When we study the behavior of Average Fraction of Rich Neighbours (*F*_R_) with decreasing *β*_move_, we expect to see a sharp transformation from a segregated to mixed wealth state, as observed in our previous work [4]. Observing blue line in Fig 4, we find two distinct regimes in *F*_R_ as the Disallowed-Realised Ratio increases. We find a slight increase in *F*_R_ as Disallowed-Realised ratio initially increases, before the sharp transformation from a segregated to a mixed-wealth configuration. It is, however, important to note that the onset of the sharp transformation is happening much later now, at a Disallowed-Realised Ratio of ∼36%, when compared to our earlier model of wealth segregation [4], where it happened when the Disallowed-Realised Ratio increased just above 0. We explore this further in our discussion on the sensitivity of the model outcomes to *β*_choice_.

Average Size of *e*_0_ Neighbourhoods (*S*_0_) also shows a non-linear sharp transformation, but from a less ethnically segregated state to a more ethnically segregated state with declining *β*_move_. Therefore, as *β*_move_ decreases, increasing the ease of movement by progressively relaxing the wealth threshold condition and thus causing a sharp decline in wealth based segregation, it simultaneously appears to increase ethnic segregation. Observing the green line in Fig 4, we notice that the onset of the sharp transformation in *S*_0_ occurs at a Disallowed-Realised Ratio of ∼36%, and peaks at a Disallowed-Realised Ratio of ∼45%. *S*_0_, therefore, monotonically increases with decreasing *β*_move_.

In summary, we find that just as disallowed-realised moves begin to occur, both wealth and ethnic segregation show concurrent, albeit slight, increases, but at a disallowed-realised ratio of ∼36% there is a sharp, non-linear transformation in opposite directions—wealth segregation rapidly declines, while ethnic segregation abruptly rises.

What our results suggest is that the dynamics of interaction between wealth and ethnicity of agents could yield opposing segregation tendencies, meaning that a highly wealth segregated configuration would correspond to low ethnic segregation, and conversely a mixed wealth configuration (low wealth segregation) would correspond to high ethnic segregation. This implies that low wealth segregation and high ethnic segregation are potentially twinned outcomes, reflecting the same state of the world.

However, before we discuss possible mechanisms causing the trade-off between segregations, we need to ascertain the robustness of this result by exploring a wider swathe of the model’s parameter space.

## Sensitivity to Model Parameters

Specifically, we check for sensitivity to the following parameters: system size (*M*) and population; correlation between wealth and ethnicity of agents; shape of wealth distribution; rate of migrant entry (*r*_mig_); difficulty of *e*_1_ agents in entering the city (*β*_in_); willingness of agents to move into lower wealth neighbourhoods (*β*_choice_); and to tolerance level of *e*_0_ agents (*τ*). For each of these analyses, we run an ensemble of five model runs.

**Fig 5:**
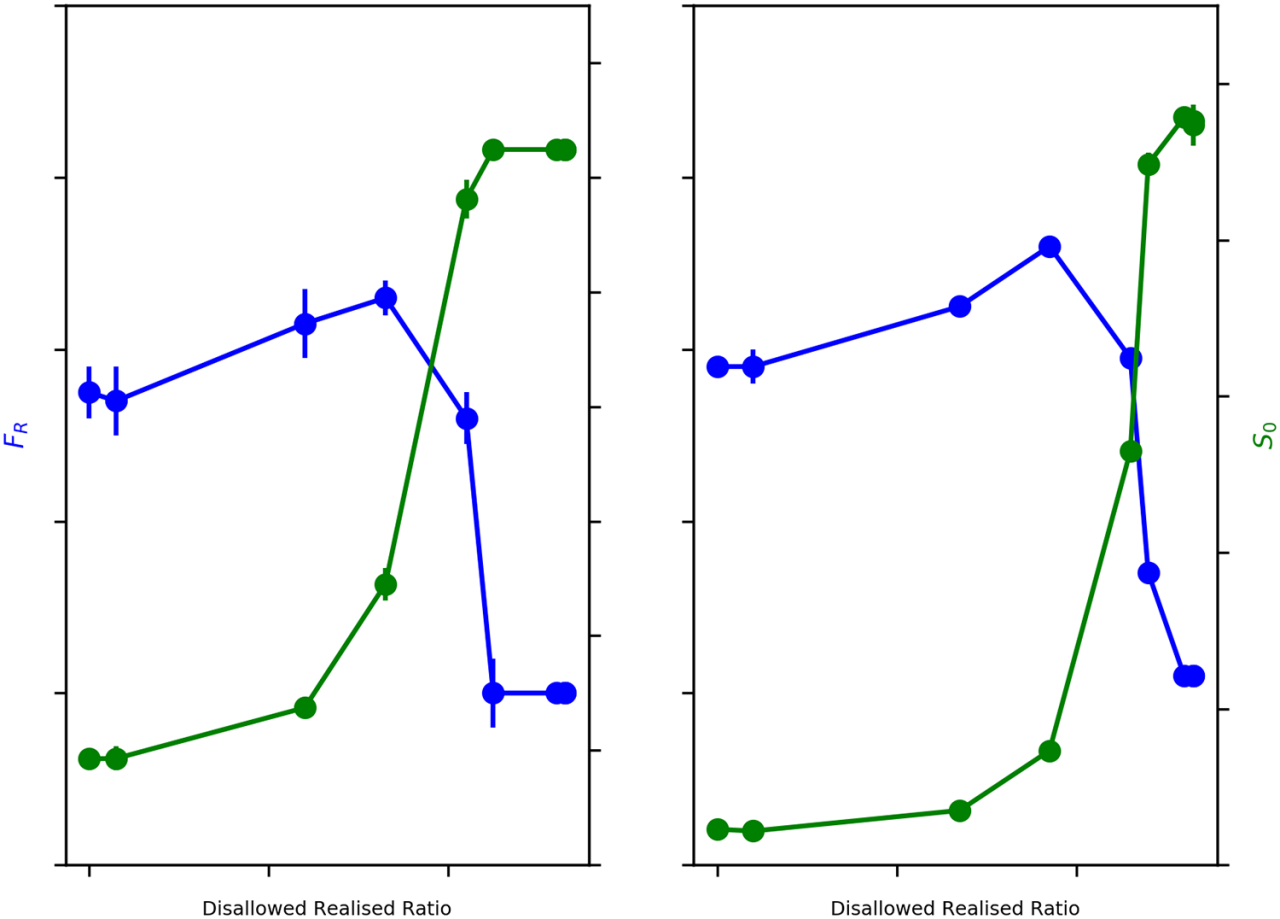
Sensitivity to system size and population. Blue: Average Fraction of Rich Neighbours (*F*_R_) v. Disallowed-Realised Ratio. Green: Average Size of *e*_0_ Neighbourhoods (*S*_0_) v. Disallowed-Realised Ratio. Left: *M* = 25 and initial population = 1,250. Right: Sensitivity to System Size: *M* = 100 and initial population = 5,000. Error bars represent 95% Confidence Interval.

We start by studying the dependence of the model dynamics on ‘city’ size and population. We simulate for smaller and bigger city sizes and populations—first with 25 neighbourhoods and 1,250 initial agents, and the with 100 neighbourhoods and 5,000 initial agents. We find that the emergence of dual segregations in opposite directions, as in the original model, obtains for both scenarios (Fig 5).

We see the same sharp decline in wealth segregation for a Disallowed-Realised ratio between 0.3 and 0.4, as well as the corresponding increase in ethnicity based segregation in both cases. This is as we would expect because the dynamics underlying the model are exactly the same, irrespective of the size of the simulation. This result suggests that model behavior is independent of system size and population.

Next, we explore the dynamics generated by varying the correlation between wealth and ethnicity of agents. In our original model, there is some correlation between ethnicity and wealth, with *e*_0_ agents, on average, having higher wealths than *e*_1_ agents (there is still significant overlap between the two wealth distributions). Now, we simulate the model for two additional scenarios—one with complete correlation between wealth and ethnicity, and the second with no correlation at all. For complete correlation, we choose agent wealths from distributions with almost zero probability of overlap: *e*_0_ agent wealths are drawn from *N*(15, 1) and *e*_1_ wealths are drawn from *N*(5, 1). For no correlation, we choose both *e*_0_ and *e*_1_ agent wealths from *N*(10, 1).

**Fig 6:**
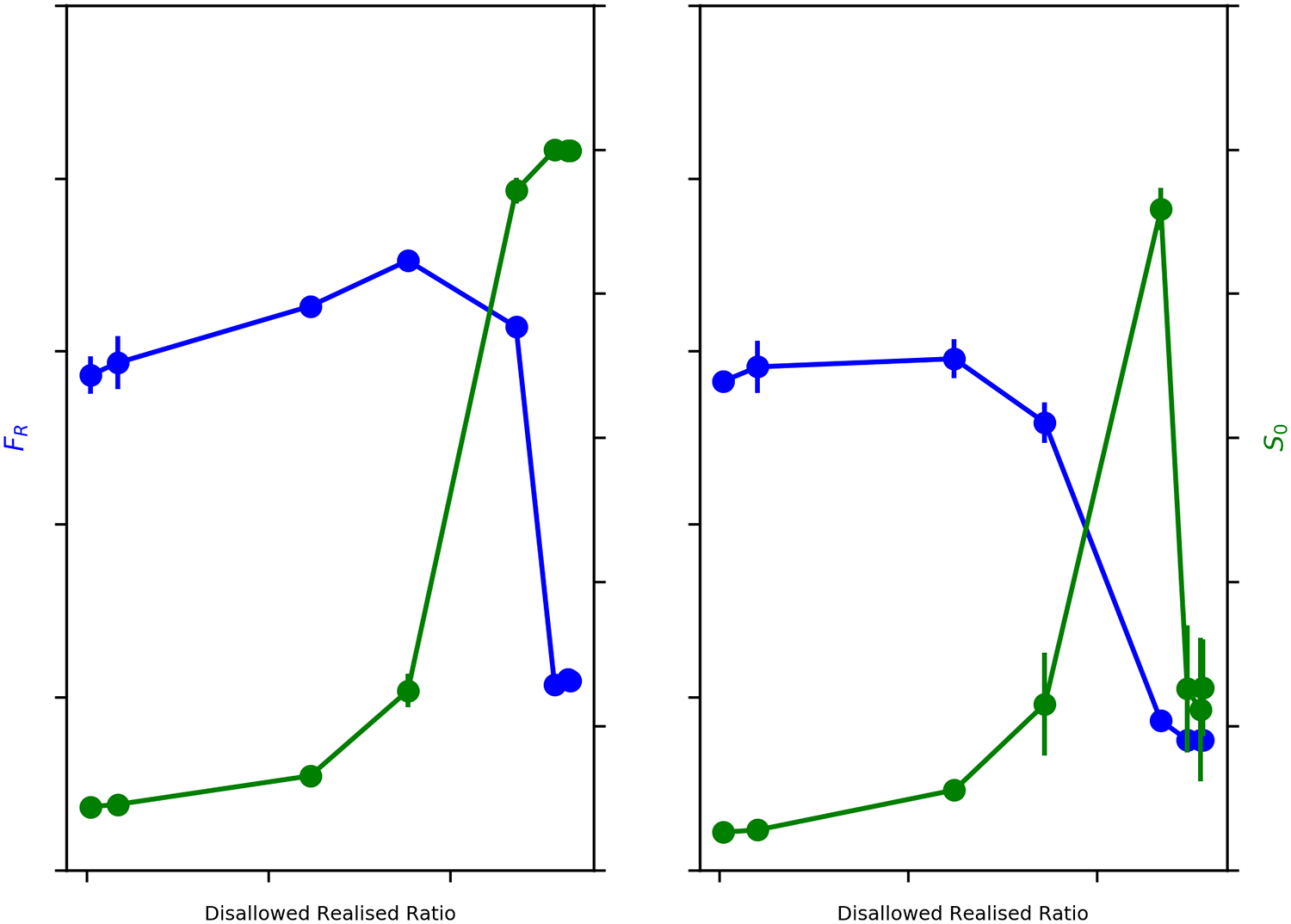
Sensitivity to correlation between agent wealth and agent ethnicity. Blue: Average Fraction of Rich Neighbours (*F*_R_) v. Disallowed-Realised Ratio. Green: Average Size of *e*_0_ Neighbourhoods (*S*_0_) v. Disallowed-Realised Ratio. Left: Wealth and ethnicity uncorrelated. Right: Wealth and ethnicity completely correlated. Error bars represent 95% Confidence Interval.

We find that for complete correlation, the model behaviour is analogous to the original model as we would expect. Fig 6 (left panel) illustrates this similarity—we see a sharp transformation from segregated to mixed wealth configurations (*F*_R_) and the corresponding transformation in the opposite direction for ethnicity-based segregation (*S*_0_).

For the no correlation scenario also, overall, the behavior of the model is analogous to that of the original model, but there is a significant decline in the values attained by *S*_0_ at very low *β*_move_, which does not occur in the original model (Fig 6, right panel). This decline in *S*_0_ can be attributed to the fact that the neighbourhood wealth threshold condition essentially becomes superfluous for *β*_move_ ≤ 0.1, thus yielding mixed-wealth configurations (as in the original model), but also now because all agents are drawn from the same wealth distribution, many more *e*_1_ agents are able to afford movement into neighbourhoods they choose (as compared to the original model), thus preventing the spatial congregation of *e*_0_ agents. However, it is important to recognise that this decline in *S*_0_ only happens at extremely high Disallowed-Realised ratios (∼50%), and that indeed the trade-off between the two segregations is already clearly demonstrated.

**Fig 7:**
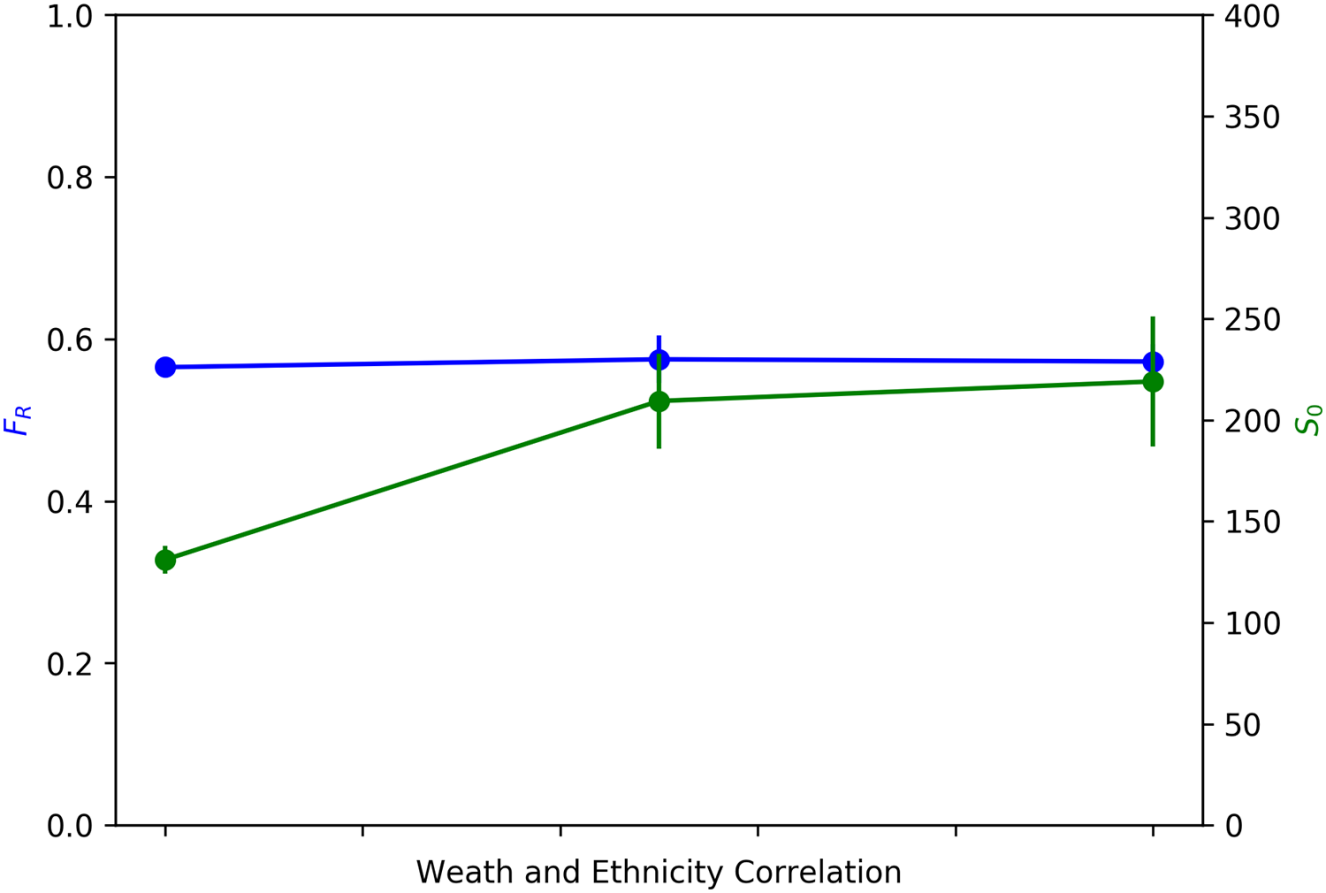
Comparison of results with Benard and Willer. Blue: Average Fraction of Rich Neighbours (*F*_R_) v. Correlation between wealth and ethnicity. Green: Average Size of *e*_0_ Neighbourhoods (*S*_0_) v. Correlation between wealth and ethnicity. Error bars represent 95% Confidence Interval.

Given this analysis of correlation between wealth and ethnicity, we also compare our results those of Benard and Willer [21]. They had found that given endogenous housing prices, both wealth and status segregation increased with increasing correlation between wealth and status. We also find that ethnic segregation increases with increasing correlation between wealth and ethnicity (Fig 7, green line), but that wealth segregation remains flat with increasing correlation (Fig 7, blue line). This difference is potentially due to the fact that in our model agent choice is dependent both on the wealth and ethnic composition of neighbourhoods, while in Bernard and Willer’s model, agent choice is contingent only upon the status composition of neighbourhoods, and not upon the wealth composition.

**Fig 8:**
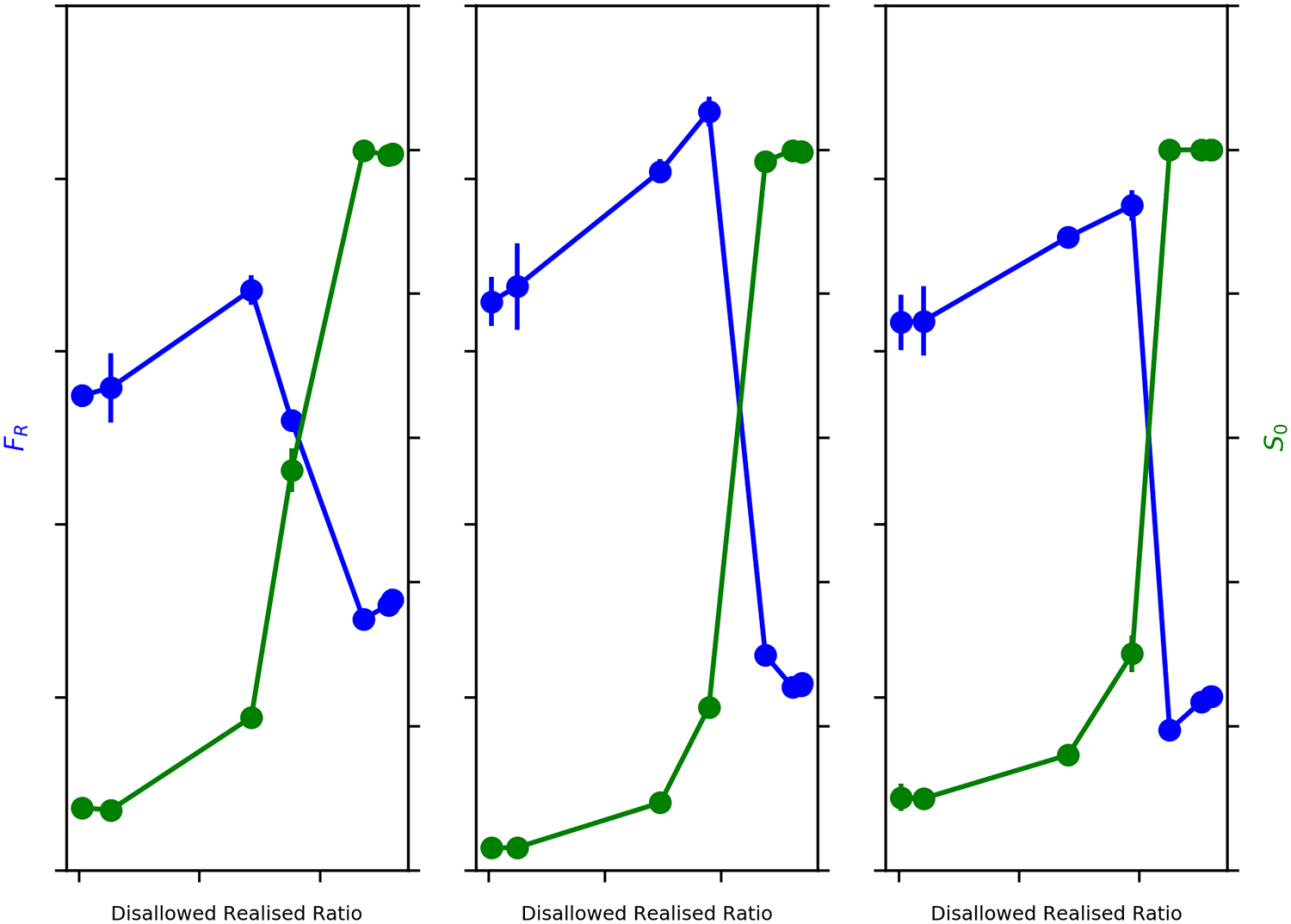
Sensitivity to shape of wealth distribution, migrant entry rate (*r*_mig_), and difficulty of migrant entry into city (*β*_in_). Blue: Average Fraction of Rich Neighbours (*F*_R_) v. Disallowed-Realised Ratio. Green: Average Size of *e*_0_ Neighbourhoods (*S*_0_) v. Disallowed-Realised Ratio. Left: *e*_0_ agents: *LN*(0.75, 0.25) and *e*_1_ agents: *LN*(0, 0.25). Centre: *r*_mig_ = 0.025 for 100 out of 500 iterations. Right: *β*_in_ = 10. Error bars represent 95% Confidence Interval.

Our original model assumes a normal distribution of wealth for agents, but real world distributions of wealth tend to be described variously by exponential, lognormal, gamma, and power-law distributions [34] [35] [36] [37]. In order to test the robustness of our model results to the shape of the wealth distribution, we now use a lognormal distribution of agent wealths—*e*_0_ agent wealths are sampled from *LN*(0.75, 0.25), and *e*_1_ agent wealths from *LN*(0, 0.25) distributions. We would expect that the outcomes of our original model are replicated, and indeed Fig 8 (left panel) confirms that the trade-off between dual segregations occurs in this scenario as well.

Next, we vary the rate of migrant (*e*_1_) entry: *r*_mig_ = 0.025 per iteration, as against 0.005 per iteration in the original model. While *r*_mig_ is five times the original model, we also vary the duration of migrant entry, allowing migrant entry only for the first 100 iterations out of the total of 500 iterations per run of the model. Urselmans’ [23] work found that the rate and size of migration had no effect on the segregation outcomes, and we would expect therefore that both wealth- and ethnic segregations exhibit the same behaviour as in the original model. This is borne out in Fig 8 (centre panel), where we see the same trade-off between *F*_R_ and *S*_0_ as in the original model.

We now move on to understanding the model dynamics, varying the difficulty of agents moving into the city (calibrated by *β*_in_). We find that the behavior of wealth-based segregation and ethnicity-based segregation is similar to that seen in the original model, as confirmed by Fig 8 (right panel). This therefore appears to suggest that the outcomes yielded by the dynamics hold irrespective of the ease (or difficulty) of *e*_1_ migrant entry into the city.

**Fig 9:**
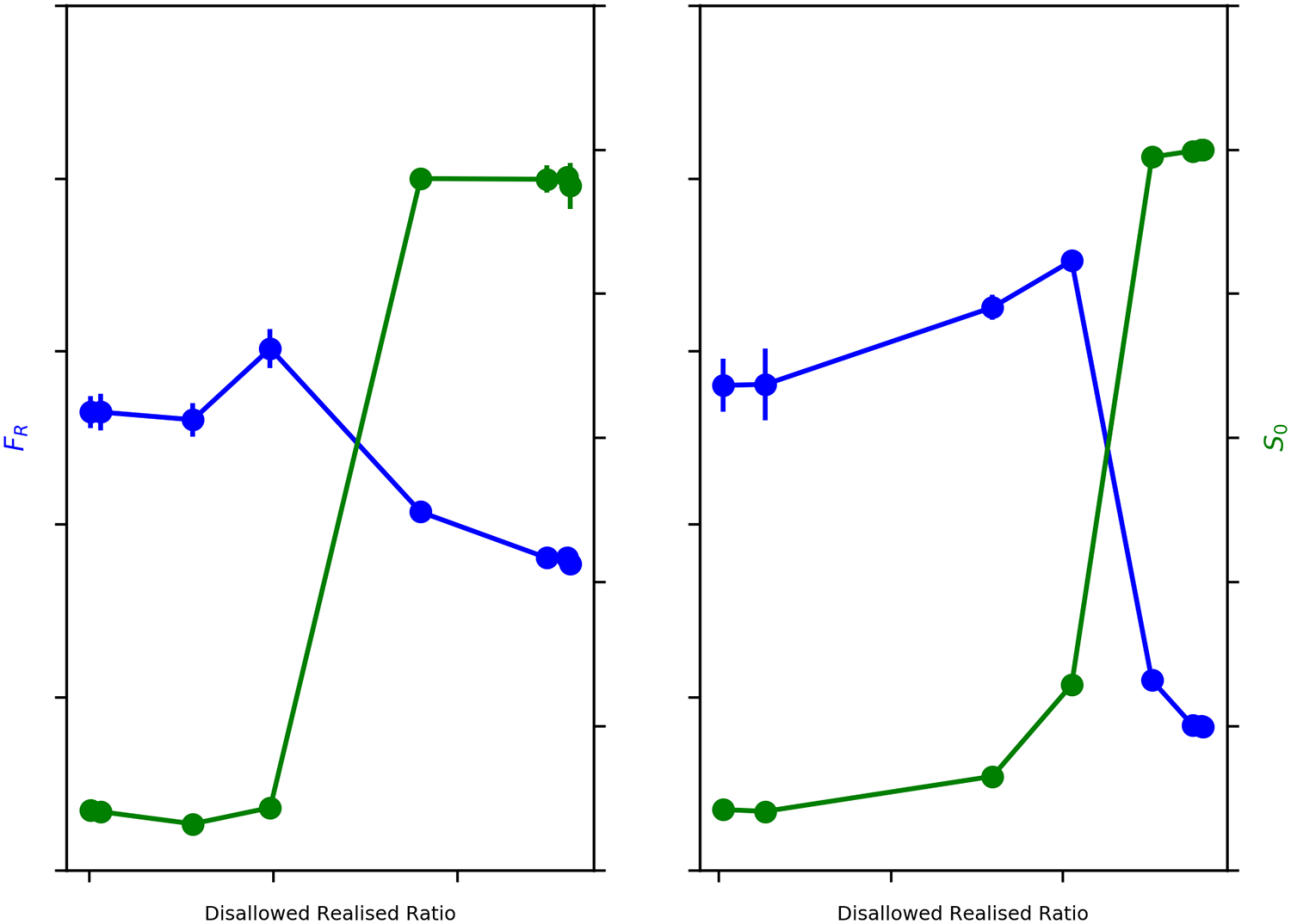
Sensitivity to willingness of agents to move into less wealthier neighbourhoods (*β*_choice_). Blue: Average Fraction of Rich Neighbours (*F*_R_) v. Disallowed-Realised Ratio. Green: Average Size of *e*_0_ Neighbourhoods (*S*_0_) v. Disallowed-Realised Ratio. Left: *β*_choice_ = 2. Right: *β*_choice_ = ∞. Error bars represent 95% Confidence Interval.

In analysing sensitivity to *β*_choice_, we find the onset of the sharp transformation from a wealth segregated to mixed wealth state occurring at a lower value of Disallowed-Realised Ratio (∼20%) for *β*_choice_ = 2 (Fig 9, left panel), and at a higher value of Disallowed-Realised Ratio (∼41%) for *β*_choice_ = ∞ (Fig 9, right panel), compared to ∼36% in the original model (*β*_choice_ = 10). Essentially, while the sharpness of transformation from segregated to mixed-wealth states is evident in all scenarios, the actual onset of the transformation requires progressively greater Disallowed-Realised moves as the propensity of agents to move out of their extant neighbourhoods in contravention of the neighbourhood comparison condition decreases. Therefore, the more agents that are willing to move in contravention of this neighbourhood comparison condition, the earlier is the onset of wealth de-segregation. Despite the difference in onset of the sharp transformation, it is apparent that the dual transformations in wealth and ethnicity themselves are, as expected, non-linear and moving in opposite directions.

**Fig 10:**
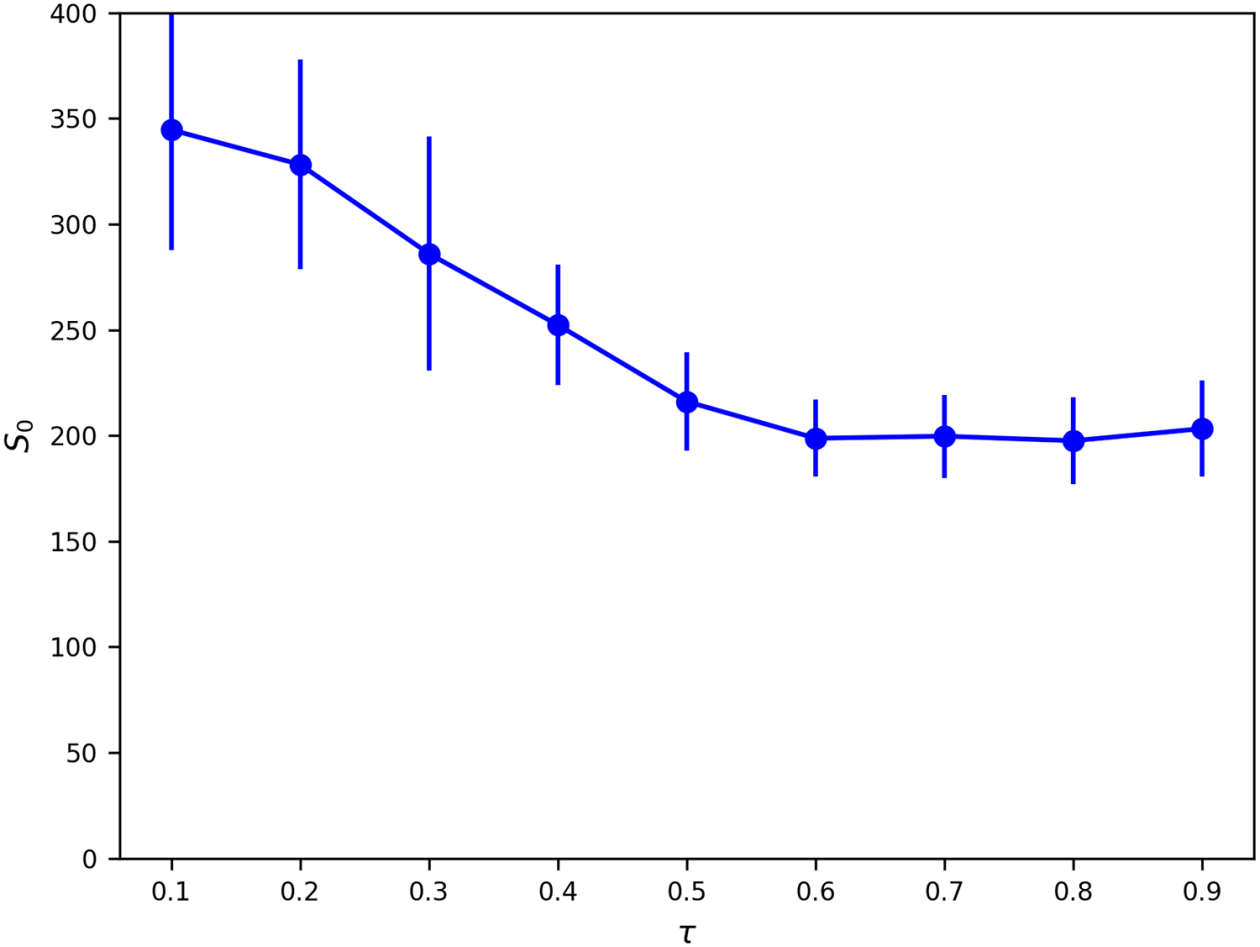
Sensitivity to agent tolerance level (*τ*). Average Size of *e*_0_ Neighbourhoods (*S*_0_) vs. Tolerance Level (*τ*). Error bars represent 95% Confidence Interval.

We also explore the dynamics generated by the model for different values of tolerance levels (*τ*) of *e*_0_ agents. We run the model with parameters detailed in Table 1 (only for *β*_move_ = 100, representing stringent neighbourhood threshold conditions to reasonably correspond with real world conditions) but for *τ* varying from 0.1 to 0.9 (0.1, 0.2, 0.3, 0.4, 0.6, 0.7, 0.8, 0.9). We would expect that as *τ* increases, *S*_0_ would decrease (even at a reasonably high *β*_move_ as is the case here) because with increasing tolerance to agents of the other ethnicity, there would be lesser ethnic clustering. Fig 10 displays this declining trend with increasing *τ*, until *τ* = 0.6, beyond which ethnicity based clustering essentially stabilizes. This is potentially due to the fact that for *τ* ≥ 0.6, all movement is determined by the neighbourhood wealth condition because such high levels of tolerance for unlike neighbours essentially inhibit the tendency for ethnicity based clustering beyond the level that which is already being caused by the wealth condition. Given the expected value of *S*_0_ for a completely mixed configuration, *S*_0_(*mix*) = 49, we see that ethnicity based clustering even for high *τ* is at a minimum 4*S*_0_(*mix*). Therefore, even in situations where *e*_0_ agents have a very high tolerance for *e*_1_ agents, and irrespective of the stringency of the wealth threshold condition, some amount of ethnicity based clustering appears inevitable.

Finally, we also compare the outcomes of our model to those of the metapopulation model of Gargiulo, Gandica, and Carletti [25]. In that model, agent movement is driven solely by ethnicity considerations (and not wealth), and it studies the outcomes on cell level ethnic heterogeneity as well as population heterogeneity. The authors find that for tolerance levels up to 0.5, cell level segregation emerges and that indeed each individual cell (neighbourhood) in the lattice is completely segregated under these scenarios. In this regime of tolerance values, they also find that cell populations tend to be heterogeneously distributed despite beginning with a homogeneous configuration. We seek to now measure these outcomes in our model and verify if these results are replicated. We use the definition of cell segregation indicator as provided by Gargiulo, Gandica, and Carletti [25]. Given that $f_{e_{0}}^{\text{i}}$ and $f_{e_{1}}^{\text{i}}$ are the fractions of *e*_0_ and *e*_1_ agents (as proportions of total *e*_0_ and total *e*_1_ agents respectively) in cell *i*, the cell segregation indicator CSI is defined as (Eq 9):

$$\begin{array}{c} \text{CSI}=\frac{1}{\sum_{i|f\left(e_{0}^{i}\right)+f\left(e_{1}^{i}\right)>0}1}\sum_{i|f\left(e_{0}^{i}\right)+f\left(e_{1}^{i}\right)>0}\frac{|f\left(e_{0}^{i}\right)-f\left(e_{1}^{i}\right)|}{f\left(e_{0}^{i}\right)+f\left(e_{1}^{i}\right)} \end{array}$$ (9)

**Fig 11:**
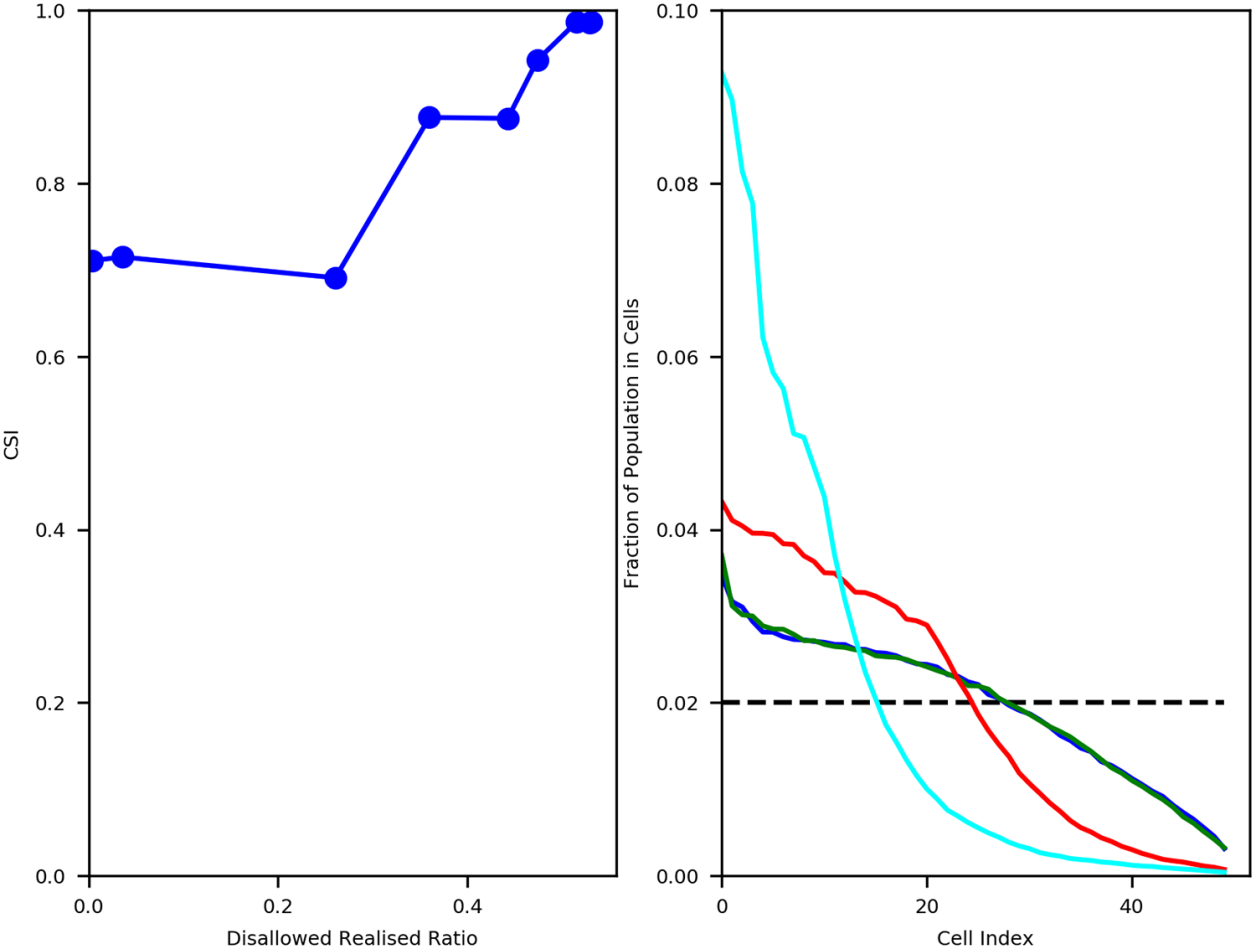
Comparison of results with Gargiulo, Gandica and Carletti. Left: Cell Segregation Indicator (*CSI*) v. Disallowed-Realised Ratio. Right: Average Population distribution across all *M* = 50 cells for different values of *β*_move_ (Fraction of total population in each cell). Black Dashed Line: Fully homogeneous distribution. Blue: *β*_move_ = 1000. Green: *β*_move_ = 100. Red: *β*_move_ = 10. Cyan: *β*_move_ = 5.

We find that for *β*_move_ ≤ 0.1, where the wealth condition becomes essentially superfluous (and therefore equivalent to the Gargiulo, Gandica and Carletti implementation [25]), CSI outcome in our model is 0.99 indicating complete ethnic segregation at the cell level, which is in agreement with the outcome of [25]. We also plot the variation in CSI with Disallowed-Realised ratio (Fig 11, left panel), and find that CSI shows a sharp non-linear increase just as in the case of our measure of ethnic segregation, *S*_0_. We also plot the average cell populations of all *M* = 50 neighbourhoods, ordered by total cell wealth, for different values of *β*_move_ (Fig 11, right panel) and find that cell populations are heterogeneously distributed for all *β*_move_, despite initial homogeneous configurations. This result also appears to be in agreement with [25]. Additionally, we find that as *β*_move_ decreases, enabling more Disallowed-Realised moves, the extent of population heterogeneity progressively increases.

Given this exploration of the parameter space, which seems to suggest that the trade-off between these dual segregations is a robust result, we now turn to investigate the rationale behind the emergence of this trade-off that enables correspondence with empirical evidence.

## Discussion

Just as there is the onset of transformation from a segregated to a mixed wealth state, we see a corresponding sharp transformation from an ethnically fragmented configuration to an increasingly ethnically segregated configuration. It is important to note that even with the ethnically fragmented configuration at high *β*_move_, when agent movement is restricted by stringent wealth conditions, some amount of ethnic clustering has nevertheless been established. A completely fragmented configuration, for instance, would yield an Average Size of *e*_0_ Neighbourhoods, *S*_0_(*mix*) = 1 = 49, which is indeed, on average, the value of *S*_0_ at the start of the simulations. For *β*_move_ ≥ 100, we see from the green line in Fig 4 that *S*_0_ is ∼212, which is ∼4.3 times *S*_0_(*mix*), reflecting the fact that there is a not insignificant amount of ethnicity-based clustering that has occurred even at this stage.

Given that *e*_0_ agents are drawn from a higher wealth distribution, their wealths are, on average, greater than those of entering *e*_1_ agents. We would therefore expect to see early on in the dynamics that the richer *e*_0_ agents, especially, are able to segregate themselves and create rich neighbourhoods, thus erecting substantial wealth thresholds preventing significant entry by *e*_1_ agents, unless they are able to afford it. But given the discrepancies in wealth between *e*_0_ and *e*_1_ agents, we would expect that the numbers of *e*_1_ agents entering these rich cells is very low and even over time their growth is not able to overshadow the population of *e*_0_ agents. We get confirmation of this when we analyse the fraction of *e*_0_ agents in neighbourhoods and find that *e*_0_ agents in the richest 20% of neighbourhoods comprise ∼75% of the total population of all *e*_0_ agents. Additionally, the fact that wealth based segregation is significant for *β*_move_ ≥ 100 implies that the *e*_0_ agents in these neighbourhoods are, on average, rich agents. *e*_1_ agents are more easily able to enter the rest of the neighbourhoods, and the entry of some *e*_1_ agents early on makes it possible over time for greater numbers of them to enter these neighbourhoods even if in contravention of the wealth threshold condition. We would therefore expect that a significant proportion of the *e*_1_ agents are in the poorer neighbourhoods. This is confirmed when we analyse the fraction of *e*_1_ agent population in the rich and poor neighbourhoods: *e*_1_ agents in the richest 20% of neighbourhoods comprise ∼56% of the total population in these neighbourhoods, which represents ∼20% of the overall *e*_1_ population; however, in the poorest half of neighbourhoods, they comprise ∼99% of the total population, with *e*_0_ agents almost completely absent from these cells.

As the first Disallowed-Realised moves begin to occur, we find that a very small number of higher wealth agents in poorer neighbourhoods are now able to contravene wealth thresholds in richer neighbourhoods (that they were unable to at higher *β*_move_) and enter them, thus resulting in the poor neighbourhoods they leave behind becoming even poorer, causing a marginal increase in *F*_R_. Also given the fact that richer *e*_0_ agents are able to segregate themselves into specific neighbourhoods early on in the dynamics, and the high barrier this places for entry by *e*_1_ agents, it is apparent that the agents able to contravene the wealth threshold condition are largely *e*_0_ agents. This means that we would expect to see a marginal increase in *S*_0_, and indeed this is what we find. In this regime, we therefore see concomitant slight increases in both *F*_R_ and *S*_0_.

As Disallowed-Realised moves further increase (with decreasing *β*_move_), and at a Disallowed-Realised Moves ratio of ∼36%, we see the sharp rise in ethnic segregation, coinciding with the onset of dramatic drop in wealth based segregation, as evidenced by Fig 4. The emergence of more mixed wealth configurations increasingly allows lower wealth *e*_1_ agents to move into neighbourhoods they were earlier unable to afford. The dynamics also allow more of the poorer *e*_0_ agents to cluster with the richer *e*_0_ agents in contravention of the neighbourhood wealth threshold condition, thus not only creating mixed wealth neighbourhoods but also increasingly large clusters of *e*_0_ agents. This argument is substantiated by the population profile of the richest 20% of neighbourhoods (for *β*_move_ ≤ 5), where we find over 99% of all *e*_0_ agents situated—thus generating the high levels of ethnic segregation. However, in contrast to the case when *β*_move_ ≥ 100, where only ∼20% of the *e*_1_ agent population was situated in the richest 20% of neighbourhoods, for *β*_move_ ≤ 5 we find that this has gone up to ∼69%—thus generating the mixed wealth configurations yielding the essential decline in wealth-based segregation.

Therefore, our analysis suggests that given a context where the two forces of wealth- and ethnicity-based spatial clustering are in effect, as agents are allowed to contravene neighbourhood wealth thresholds, we would expect both wealth and ethnic segregations to marginally increase initially and then show sharp, non-linear transformation in opposite directions—wealth-based segregation breaks down and yields mixed wealth configurations, while ethnic segregation rapidly worsens. In Schelling’s original model [6], we see the emergence of very high levels of racial segregation (81.5%) at agent tolerance level = 0.5, in the absence of a wealth threshold. This leaves us with the conclusion that the restrictions on movement imposed by neighbourhood wealth thresholds (at high *β*_move_) indeed controls the extent of ethnic segregation, which in the absence of these restrictions could be substantially higher. Additionally, we can also infer that when agents have even a slight preference for co-ethnics, it is practically impossible to get completely mixed ethnic configurations and that some amount of ethnic segregation is inevitable, irrespective of the stringency of the wealth threshold condition.

This analysis also indicates that the mixed wealth configurations created by increasing Disallowed-Realised moves directly enable the corresponding sharp transformation from a less ethnic segregated to highly ethnic segregated city. We can think of the highly mixed wealth configurations as scenarios where neighbourhood wealth thresholds essentially become superfluous (*β*_move_ ≤ 0.1) and it is in these cases we see the full extent of possible ethnic segregation because *e*_0_ agents congregate in neighbourhoods they prefer with practically no wealth restrictions on them. It is therefore the case the very movement of agents that allow for decreased wealth segregation, ends up enabling the exacerbation of ethnic segregation.

Given this trade-off posited by our theoretical model, we turn to the available empirical evidence on dual segregation. While there are no studies that directly compare the extent of wealth- and ethnicity- based segregations, there has been significant research on the socioeconomic effects of economic segregation as well as ethnic fragmentation as distinct phenomena [38] [39] [40] [41] [42] [43] [44] [45] [46] [47] [48].

There is a rich body of evidence from the economics and political science literature on the impact of wealth segregation on an array of socioeconomic outcomes [38] [39] [40] [41] [42]. Educational outcomes of youth from poorer households are found to be negatively impacted by economic segregation, with lower rates of high school graduation observed for poorer adolescents [38]. Economically mixed and middle income cities are shown to demonstrate significantly higher levels of civic participation than economically segregated cities, on account of greater competition for public goods that encourages citizen interest and participation in economically mixed cities [39]. The risk of mortality especially among, but not restricted to, the poor is also significantly associated with increasing socioeconomic segregation [40]. In addition, neighbourhoods have been found to affect inter-generational mobility, with outcomes of children moving into a better neighbourhood improving linearly with the time spent in that area [41]. Living in higher income neighbourhoods is found to be a significant and positive determinant of future incomes of children from lower income households. There is also a significant positive impact on employment and income levels of children moving out of segregated neighbourhoods [42]. Summarily, the evidence suggests that lowering wealth segregation has direct, positive outcomes on multiple socioeconomic outcomes such as education, health, civic participation, income, and employment.

A parallel strand of empirical work from sociology examining homophily presents us with evidence of socioeconomic impacts on account of ethnic segregation. Homophily is the tendency of individuals to bond with others who are similar, and the homophily of ethnicity is found to create the strongest divides in personal environments [43]. There is, however, overwhelming evidence from around the world that increasing ethnic fragmentation (implying increased spatial mixing between ethnicities or low ethnic segregation) negatively impacts the provision of public goods in both urban and rural contexts [44] [45] [46] [47] [48]. In their analysis of data on a wide range of public goods such as educational institutions, health care facilities, water sources, and transport and communication infrastructure in rural India, Banerjee, Iyer and Somanathan [44] find that areas with high levels of caste-based fragmentation had lower access to these public goods. A study of American cities finds that productive public goods like education, roads, libraries, sewers and trash pickup are inversely related to the level of the city’s ethnic fragmentation [45]. The authors argue that ethnic fragmentation is negatively related to the share of social spending on welfare because it implies difference in tastes that cause disagreements about priorities, resulting in underinvestment and underprovision of public assets. It has similarly been argued that co-ethnics share a common reservoir cultural material such as language and experience that makes it easier for them to work together [46]. Miguel and Gugerty [47] find that in rural Kenya ethnic fragmentation is associated with lower school funding and worse school facilities, which they attribute to the collective action failures resulting out of the inability of such communities to impose sanctions. Successful public goods provision in ethnically segregated communities is also observed in Uganda [48], which the authors attribute to closer linkages through social networks and thus the credible threat of social sanctions. These findings support the contention of Banerjee, Iyer and Somanathan [44] that the hypothesis of social fragmentation undermining economic progress is amongst the most powerful in political economy.

Overall, the empirical evidence from these strands of inquiry suggests the possibility of a trade-off between wealth segregation and ethnic segregation. However, it is important to stress here that this relationship is only implied by bringing together studies that have considered the wealth and ethnicity effects separately, not together and in the same context. Therefore, it will require further empirical work to ascertain the true nature of the relationship between wealth segregation and ethnic segregation, and to conclusively verify the potential trade-off between these dual segregation tendencies.

## Conclusion

We build a model incorporating wealth and ethnicity to explore the emergence of and interplay between ethnic and wealth-based segregations. We find that as agents are progressively allowed to contravene neighbourhood wealth thresholds, initially there is a marginal increase in both wealth and ethnic segregations, followed by a sharp transformation in opposite directions—a drop in wealth segregation accompanied by a rise in ethnic segregation. We argue that it is the mixed wealth configurations that are enabled by an easing of the neighbourhood wealth threshold condition, which provide the impetus for increased ethnic segregation. Therefore, our work posits that a decrease in wealth segregation does not merely accompany, but in fact drives, the increase in ethnic segregation as neighbourhood wealth thresholds are progressively contravened.
